# Loss of Fbxw7 disrupts lipid homeostasis and autophagy in hepatocellular carcinoma cells

**DOI:** 10.1007/s00795-026-00457-3

**Published:** 2026-03-26

**Authors:** Yoshihiro Hayashi, Yumiko Yamamoto, Ichiro Murakami

**Affiliations:** 1 Department of Pathology, School of Medicine, Kochi University, 185-1, Kohasu, Oko-cho, Nankoku, 783-8505 Japan; 2 Science Research Center, Kochi University, 185-1, Kohasu, Oko-cho, Nankoku, 783-8505 Japan

**Keywords:** Fbxw7, Hepatocellular carcinoma, Lipid droplets, Autophagy, SREBP-1, Morphological degeneration

## Abstract

Fbxw7, a substrate recognition subunit of the SCF ubiquitin ligase complex, regulates the proteasomal degradation of multiple cancer-related and metabolic proteins. To elucidate its role in hepatic lipid homeostasis, we established Fbxw7 knockdown (FKD) Huh-7 cells and analyzed associated morphological and molecular alterations. Lipid droplets were evaluated by BODIPY staining and transmission electron microscopy, while metabolic and autophagy-related factors were examined using immunocytochemistry, Western blotting, and RT-PCR. FKD resulted in marked lipid droplet accumulation and upregulation of lipogenic genes, accompanied by increased levels of both precursor and mature sterol regulatory element-binding protein 1 (SREBP-1) with prominent nuclear localization. In three-dimensional spheroid cultures, FKD cells exhibited extensive vacuolar degeneration, reduced LC3B expression, and p62 accumulation, whereas LAMP-1 expression remained unchanged. Low-vacuum scanning electron microscopy further revealed rough, granular deposits on the spheroid surface, suggesting structural deterioration associated with impaired autophagy. Treatment with the autophagy inducer Tat-Beclin1 partially restored LC3B expression and attenuated lipid droplet accumulation. Collectively, these findings indicate that Fbxw7 plays a critical role in maintaining hepatocellular homeostasis through coordinated regulation of lipid metabolism and autophagic degradation. Loss of Fbxw7 disrupts this balance, leading to characteristic metabolic and morphological alterations in hepatocellular carcinoma cells.

## Introduction

In recent decades, changes in dietary habits and lifestyle patterns characterized by increased caloric intake and reduced physical activity have led to a rising prevalence of metabolic syndrome and a marked increase in overnutrition-related fatty liver disease, now affecting nearly 30% of the global population [1]. Although metabolic dysfunction associated steatotic liver disease (MASLD) was long considered benign in individuals without alcohol intake, it is now recognized that a subset of these patients can progress to chronic hepatitis, cirrhosis, and hepatocellular carcinoma (HCC) through the intermediate stage of metabolic dysfunction-associated steatohepatitis (MASH) [2]. Despite substantial advances, the molecular mechanisms governing lipid droplet dynamics and hepatocarcinogenesis remain incompletely understood.

Emerging evidence indicates that autophagosomes can engulf lipid droplets and degrade them through lysosomal lipase activity—a selective form of autophagy referred to as lipophagy [3, 4]. Hepatic fat accumulation, once primarily attributed to caloric excess, may therefore also result from impaired lipophagy [3, 4]. Although lipophagy is increasingly recognized as a central regulator of hepatic lipid homeostasis and MASLD progression, its molecular control mechanisms have not been fully elucidated.

In this study, we aimed to clarify the role of lipophagy in hepatic lipid metabolism by employing a lipid-accumulating HCC model generated through Fbxw7 knockdown. To better recapitulate in vivo tumor architecture and metabolic features, we used a three-dimensional (3D) spheroid culture system, which more closely reflects native tissue organization and physiological behavior than traditional two-dimensional (2D) monolayers [5]. Using this model, we examined lipid droplet dynamics and associated molecular alterations to determine how Fbxw7 deficiency contributes to lipophagic dysfunction and metabolic dysregulation in hepatocellular carcinoma.

## Materials and methods

### Establishment of Fbxw7‑knockdown Huh‑7 cells

The human hepatocellular carcinoma cell line Huh‑7 (Health Science Research Resources Bank, Osaka, Japan) was cultured at 37 °C in Dulbecco’s Modified Eagle Medium (DMEM; Sigma‑Aldrich, St. Louis, MO, USA) supplemented with 10% fetal bovine serum (FBS), 100 U/mL penicillin, and 100 µg/mL streptomycin (Sigma‑Aldrich).

For stable knockdown of Fbxw7, a pRS plasmid vector carrying an shRNA targeting the Fbxw7 gene (TI318157; OriGene, Rockville, MD, USA) was transfected into Huh‑7 cells using X-tremeGENE9 Transfection Reagent (Roche Diagnostics, Basel, Switzerland) according to the manufacturer’s instructions. The vector contained a puromycin‑resistance gene, allowing for selection with 1.2 µg/mL puromycin (Sigma‑Aldrich) for two weeks. The resulting cells were designated as Fbxw7‑knockdown (FKD) Huh‑7 cells.

Control (mock) Huh‑7 cells were generated by transfecting the empty pRS vector (TR20003; OriGene) without shRNA. All experiments were performed using ten independent clones of FKD and mock Huh‑7 cells, with representative data shown in the figures.

### Immunocytochemistry and BODIPY staining

For immunocytochemical staining, FKD and mock cells were incubated with a rabbit polyclonal anti‑Fbxw7 antibody (1:200; Abcam, Cambridge, UK), followed by incubation with fluorescein isothiocyanate (FITC)‑conjugated anti‑mouse IgG secondary antibody (1:200; Molecular Probes, Eugene, OR, USA). Nuclei were counterstained with 4′,6‑diamidino‑2‑phenylindole (DAPI; Sigma‑Aldrich).

BODIPY staining (Thermo Fisher Scientific, Waltham, MA, USA) was performed according to the manufacturer’s protocol. Briefly, cells cultured in 100‑mm dishes (Iwaki Co., Ltd., Tokyo, Japan) were trypsinized, centrifuged, and pelleted. They were then fixed with 4% paraformaldehyde (Nacalai Tesque, Kyoto, Japan) at 4 °C for 4 h, embedded in Tissue‑Tek O.C.T. Compound (Sakura Finetek Japan Co., Ltd., Tokyo, Japan), and frozen Sect. (6 μm) were prepared using a cryostat (Leica Microsystems, Wetzlar, Germany). Fluorescence images were obtained using an Olympus BX53 microscope (Olympus, Tokyo, Japan).

### Western blot analysis

Cells were lysed using the Lysopure Nuclear and Cytoplasmic Extractor Kit (Fujifilm Wako Pure Chemical Corp., Osaka, Japan). Protein concentrations were determined with the Pierce BCA Protein Assay Kit (Thermo Fisher Scientific). Equal amounts of protein (20 µg/lane) were separated on 10% SDS‑PAGE gels (Bio‑Rad Laboratories, Hercules, CA, USA) and transferred onto PVDF membranes using the Trans‑Blot Turbo Transfer System (Bio‑Rad Laboratories). Antibodies and other chemical reagents are listed in Table 1.

**Table 1:** Primary antibodies, secondary antibodies and chemical agents used in immunohistochemistry

Antibody	Source	Dilution
Anti-Fbxw7 rabbit polyclonal antibody	Abeam (Cambridge, UK)	1:500
Anti-p62/SQSTM1 mouse monoclonal antibody	Abeam (Cambridge, UK)	1:1000
Anti-LAMP1 rabbit monoclonal antibody	CST (Danver, MA, USA)	1:1000
Anti-LC3B rabbit polyclonal antibody	MBL (Centennial, USA)	1:500
Anti- SREBP-1 mouse monoclonal antibody	Santa Cruz Biotechnology (Santa Cruz, CA)	1:500
Anti-Cathepsin D mouse monoclonal antibody	Calbiochem (Carpinteria, CA., USA)	1:1000
Anti-β-actin mouse monoclonal antibody	Sigma Aldrich (St. Louis, MO, USA)	1:5000
Anti-LaminA/C mouse monoclonal antibody	Abeam (Cambridge, UK)	1:1000
FITC-labeled anti-mouse IgG antibody	Molecular Probes (Eugene, OR., USA)	1:200
FITC-labeled anti-rabbit IgG antibody	Molecular Probes (Eugene, OR., USA)	1:200
Texas-Red labeled anti-mouse IgG antibody	Molecular Probes (Eugene, OR., USA)	1:200

Chemical agent	Source
4′,6-diamidino-2-phenylindole: DAPI	Sigma Aldrich (St. Louis, MO, USA)
TAT-Bcclin1 peptide: NBP2-49888	Novus Biologicals (Centennial, CO, USA)

Membranes were blocked with Blocking One (Nacalai Tesque) for 1 h at room temperature and incubated overnight at 4 °C with primary antibodies against Fbxw7, LAMP-1, p62/SQSTM1, LC3B, Cathepsin D, SREBP‑1, Lamin A/C or β‑actin. After washing, membranes were incubated for 1 h at room temperature with HRP‑conjugated anti‑mouse or anti‑rabbit secondary antibodies (Bio‑Rad Laboratories). Protein bands were visualized using ECL Prime Western Blotting Detection Reagents (Cytiva, Marlborough, MA, USA) and imaged with an LAS‑4000 Lumino Image Analyzer (Fujifilm Wako). Band intensities were quantified with ImageJ software (NIH, Bethesda, MD, USA).

### Semiquantitative RT‑PCR

Semiquantitative RT‑PCR was employed in this study because our primary objective was to compare relative gene expression levels between mock and FKD cells rather than to obtain absolute quantification. This approach is widely used for detecting expression differences in metabolic and autophagy‑related pathways and was sufficient for evaluating the relative changes observed in our experimental system. Total RNA was isolated from cells at approximately 80% confluence. cDNA synthesis and subsequent PCR amplification were performed as previously described [6]. PCR products were separated on 7% native polyacrylamide gels, stained with ethidium bromide, and visualized under UV illumination. Band intensities were quantified by densitometry using ImageJ.

Target genes included SREBP‑1, SCD, ACC, and MTP, with GAPDH serving as an internal control. Primer sequences, expected product sizes, and cycle numbers are listed in Table 2. The number of PCR cycles for each gene was optimized to ensure amplification within the linear range.

**Table 2:** Primers and cycle numbers used in semiquantitative RT-PCR

Gene	Primer sequence (5′-3′)	Product size	PCR cycle number
*SREBP-lc*	CGGAACCATCTTGGCAACAGT CGCTTCTCAATGGCGTTGT	170	29
*SCD*	TTCCTACCTGCAAGTTCTACACC CCGAGCTTTGTAAGAGCGGT	202	27
*ACC*	ATGTCTGGCTTGCACCTAGTA CCCCAAAGCGAGTAACAAATTCT	215	32
*MTP*	ACAAGCTCACGTACTCCACTG TCCTCCATAGTAAGGCCACATC	261	27
*GAPDH*	ACCACAGTCCATGCCATCAC TCCACCACCCTGTTGCTGTA	452	25
*FBXW7*	AAAGAGTTGTTAGCGGTTCTCG CCACATGGATACCATCAAACTG	236	27

### Transmission electron microscopy (TEM)

FKD and mock spheroids were fixed in 2.5% glutaraldehyde in 0.1 M phosphate buffer (PB, pH 7.4) for 24 h at 4 °C, followed by post-fixation in 1% osmium tetroxide in PB for 1 h at 4 °C. Samples were dehydrated through a graded ethanol series and embedded in epoxy resin 812 (TAAB Laboratories, Reading, UK). Ultrathin sections were stained with uranyl acetate and lead citrate and examined using a JEM‑1400 Plus electron microscope (JEOL, Tokyo, Japan).

### Spheroid culture of Huh‑7 cells

FKD and mock cells (1 × 10^4^ cells/mL) were seeded in PrimeSurface 96‑well ultra‑low‑adhesion round‑bottom plates (Sumitomo Bakelite Co., Ltd., Tokyo, Japan). After incubation at 37 °C with 5% CO_2_ for 3–7 days, multicellular spheroids were formed. Spheroids were fixed in 4% paraformaldehyde and subjected to hematoxylin and eosin (H&E) and immunohistochemical staining. Images were captured using an Olympus BX53 microscope. LV‑SEM observation was also performed as previously described [7].

### Low‑vacuum scanning electron microscopy (LV‑SEM)

FKD and mock cells or spheroids were fixed in 2.5% glutaraldehyde in 0.1 M phosphate buffer (pH 7.4) for 4 h at 4 °C and post-fixed in 1% osmium tetroxide for 1 h. After washing with distilled water, samples were stained with 1% phosphotungstic acid (Muto Pure Chemicals Co., Ltd., Tokyo, Japan) for 10 min, washed again, and air‑dried on conductive carbon adhesive tape (Nisshin EM Co., Ltd., Tokyo, Japan). Samples were examined using a JSM‑6010LV scanning electron microscope (JEOL, Tokyo, Japan).

### Treatment of spheroids with Tat‑Beclin1 peptide

After spheroid formation, the autophagy‑inducing Tat‑Beclin1 peptide (NBP2-49888; Novus Biologicals, Centennial, CO, USA) was added to the culture medium at a final concentration of 20 µM on the following day. On day 6, spheroids were fixed in 4% paraformaldehyde, embedded in paraffin, and sectioned for H&E staining.

For immunohistochemical analysis, paraffin sections were deparaffinized, rehydrated, and subjected to antigen retrieval in Immunosaver (Nisshin EM Co., Ltd.) at 98 °C for 30 min. After blocking with 5% normal goat serum, sections were incubated overnight at 4 °C with primary antibodies against LC3B (1:100; Dako) and p62/SQSTM1 (1:200; Abcam). After washing with PBS, the sections were incubated with FITC-labeled anti-rabbit IgG antibody or FITC-labeled anti- mouse IgG antibody (Molecular Probes) at room temperature for 1 h and washed again with PBS. Nuclei were counterstained with DAPI (Sigma‑Aldrich). Fluorescence images were obtained using an Olympus BX53 microscope (Olympus).

### Statistical analysis

No formal statistical hypothesis testing was performed in this study. All experiments were independently repeated three times, and band intensities from Western blotting and semiquantitative RT‑PCR were quantified using ImageJ. Data are presented as mean ± standard deviation (SD). Because the purpose of this study was to compare relative differences between mock and FKD cells, descriptive comparisons of the quantified values were used without applying additional statistical tests.

## Results

### Establishment of FKD cells

**Fig. 1:**
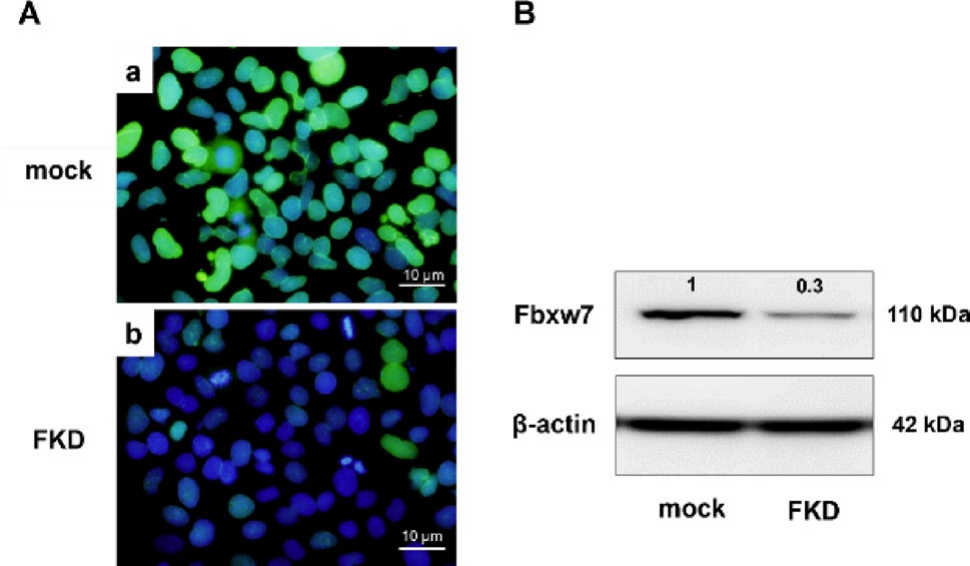
Establishment and validation of Fbxw7 knockdown (FKD) in Huh-7 cells. **A** Immunofluorescence staining demonstrated a marked reduction of Fbxw7 expression in FKD cells (**b**) compared with mock control cells (**a**). Nuclei were counterstained with DAPI. **B** Western blot analysis confirmed decreased Fbxw7 protein levels in FKD cells, validating the successful establishment of stable Fbxw7-deficient Huh-7 cell lines

Stable FKD cells were successfully generated through pRS-shFbxw7 transfection followed by puromycin selection. Immunocytochemistry demonstrated reduced nuclear Fbxw7 staining in FKD cells (Fig. 1A). Western blotting further confirmed a marked reduction in Fbxw7 protein levels in FKD cells compared with mock controls (Fig. 1B).

### Morphological changes and lipid droplet accumulation

**Fig. 2:**
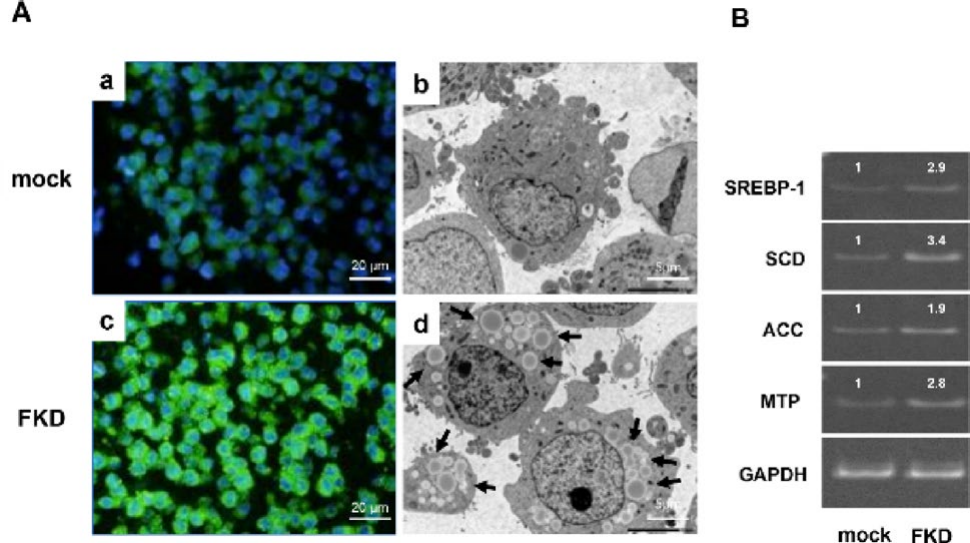
Lipid droplet accumulation and ultrastructural changes in FKD cells. **A** BODIPY staining revealed markedly increased cytoplasmic lipid droplets in FKD cells (**c**) compared with mock cells (**a**). Transmission electron microscopy (TEM) demonstrated numerous large lipid droplets and distorted cytoplasmic organelles in FKD cells (**d**) compared with mock cells (**b**). The arrows indicate lipid droplets within the cytoplasm. **B** Semiquantitative RT-PCR analysis showed upregulation of lipid metabolism–related genes (SREBP-1, SCD, ACC, MTP), indicating activation of lipogenic pathways following Fbxw7 depletion

FKD cells exhibited pronounced morphological alterations. BODIPY staining showed extensive accumulation of cytoplasmic lipid droplets, with markedly higher intensity than in mock cells (Fig. 2a, c). TEM analysis revealed abundant enlarged lipid droplets and electron-lucent vacuoles in FKD cells (Fig. 2b, d). In addition, semiquantitative RT-PCR demonstrated significant upregulation of lipogenic genes associated with lipid droplet synthesis in FKD cells (Fig. 2B).

### Expression of lipid metabolism–related molecules

**Fig. 3:**
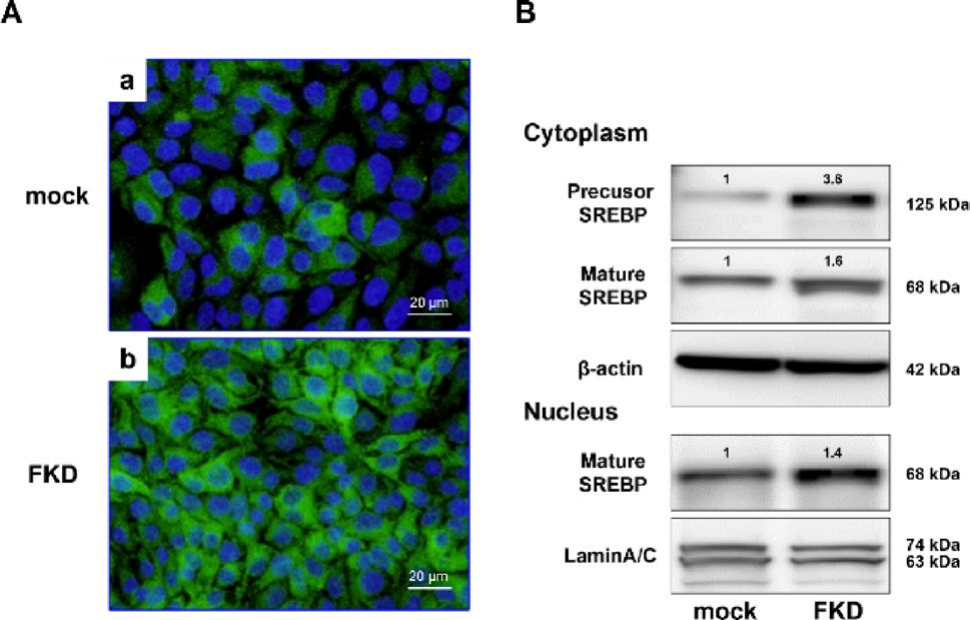
Increased SREBP-1 expression in FKD cells. **A** Immunocytochemistry showed enhanced cytoplasmic and nuclear localization of SREBP-1 in FKD cells (**a**, **b**). **B** Western blotting demonstrated increased levels of both precursor and mature forms of SREBP-1, consistent with enhanced SREBP-1 activation in FKD cells

Immunocytochemistry for SREBP-1 revealed increased expression in both the nucleus and cytoplasm of FKD cells (Fig. 3A). Western blot analysis showed a marked increase in cytoplasmic precursor SREBP-1 as well as elevated mature SREBP-1 protein. Increased nuclear accumulation of mature SREBP-1 was also confirmed (Fig. 3B).

### Morphological changes in spheroid cultures

**Fig. 4:**
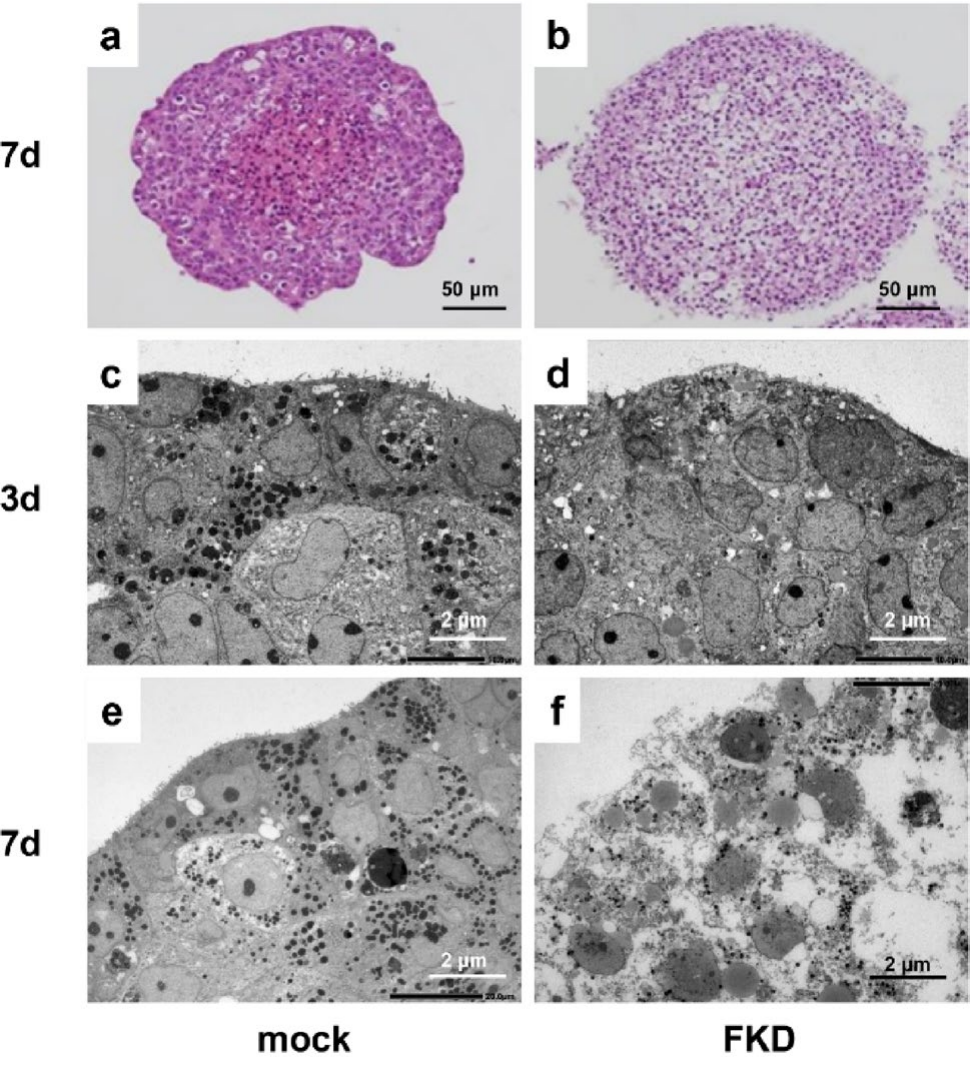
Vacuolar degeneration in FKD spheroids. Hematoxylin and eosin (H&E) staining of three-dimensional spheroid cultures revealed extensive vacuolar degeneration and partial central necrosis in FKD spheroids by day 7, whereas mock spheroids maintained compact cellular architecture (**a**, **b**). TEM images of spheroids at day 3 (**c**, **d**) and day 7 (**e**, **f**). Mock spheroids (**c**, **e**) exhibited numerous electron-dense lysosomes at both time points. FKD spheroids (d, f) showed fewer lysosomes at day 3 and marked vacuolar degeneration by day 7

In three-dimensional culture, FKD spheroids showed marked structural deterioration by day 7, with prominent cytoplasmic vacuolization observed on H&E staining (Fig. 4a, b). TEM analysis demonstrated numerous lysosomes in mock spheroids at both days 3 and 7 (Fig. 4c, e), whereas FKD spheroids displayed pronounced cytoplasmic vacuolation by day 7 (Fig. 4d, f).

### Altered autophagy-related protein expression

**Fig. 5:**
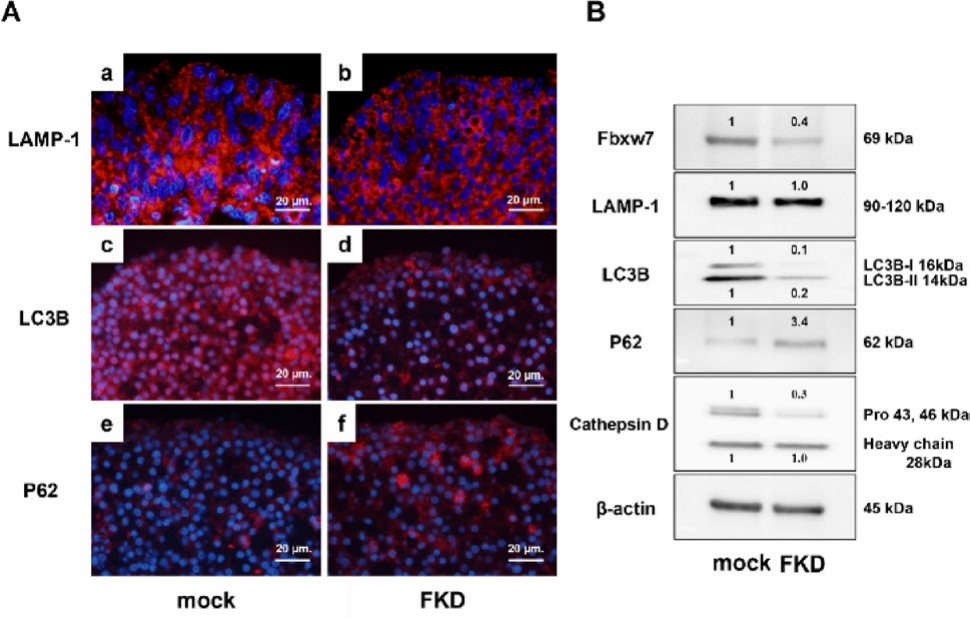
Altered expression of autophagy-related markers in FKD cells. Immunofluorescence revealed reduced LC3B staining (**c**, **d**) and increased p62 staining (**e**, **f**) in FKD cells compared with mock cells. Western blotting confirmed decreased LC3B-II and increased p62 protein levels, whereas LAMP-1 expression remained unchanged (**a**, **b**), indicating impaired autophagic flux in FKD cells

Immunocytochemistry revealed a marked decrease in LC3B expression in the FKD group, while LAMP-1 expression remained unchanged. In contrast, p62 expression was increased (Fig. 5A a–f). Western blotting confirmed reduced LC3B-I and -II, unchanged LAMP-1, and elevated p62 levels, together with decreased expression of the lysosomal protease cathepsin D (pro-form) (Fig. 5B).

### LV-SEM observation of spheroid surfaces

**Fig. 6:**
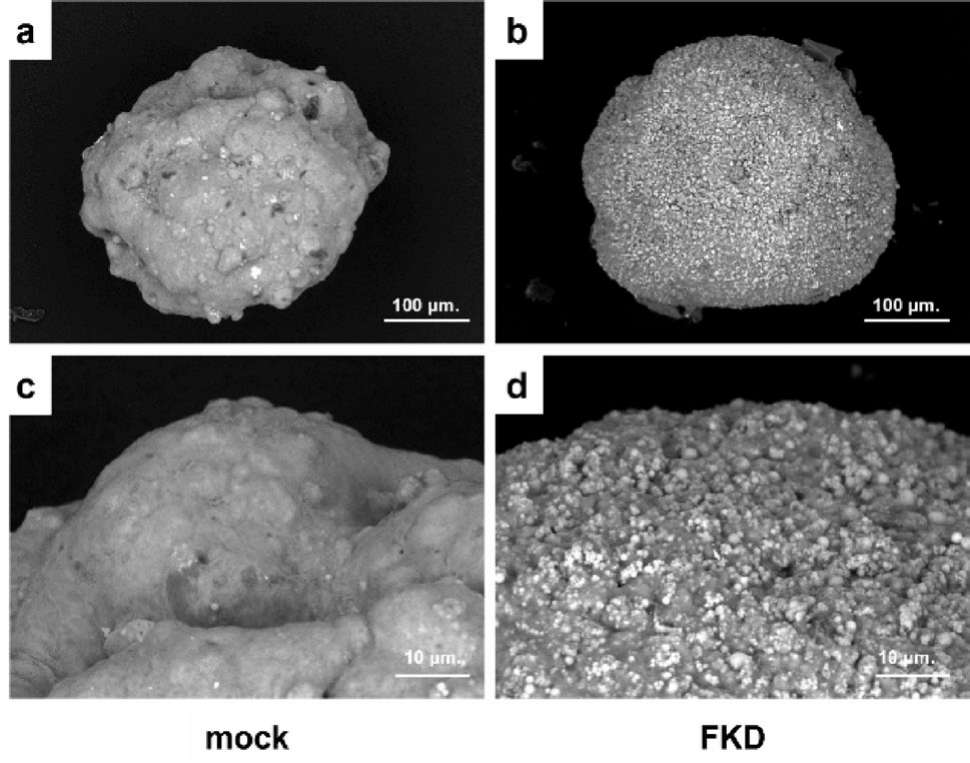
Surface ultrastructure of spheroids observed by low‑vacuum scanning electron microscopy (LV‑SEM). Representative images of mock and FKD spheroids are shown Magnifications: ×200 (a, b) and ×1,000 (c, d) FKD spheroids exhibited rough, granular surface structures compared with the smooth and compact surfaces of mock spheroids

LV-SEM imaging on day 7 after spheroid formation showed that mock spheroids maintained smooth, compact surface morphology, whereas FKD spheroids exhibited distorted spheres with irregular, granular surface texture (Fig. 6a–d). FKD spheroids were covered with fine particulate material consistent with structural degeneration.

#### Effects of Tat-Beclin1 peptide

**Fig. 7:**
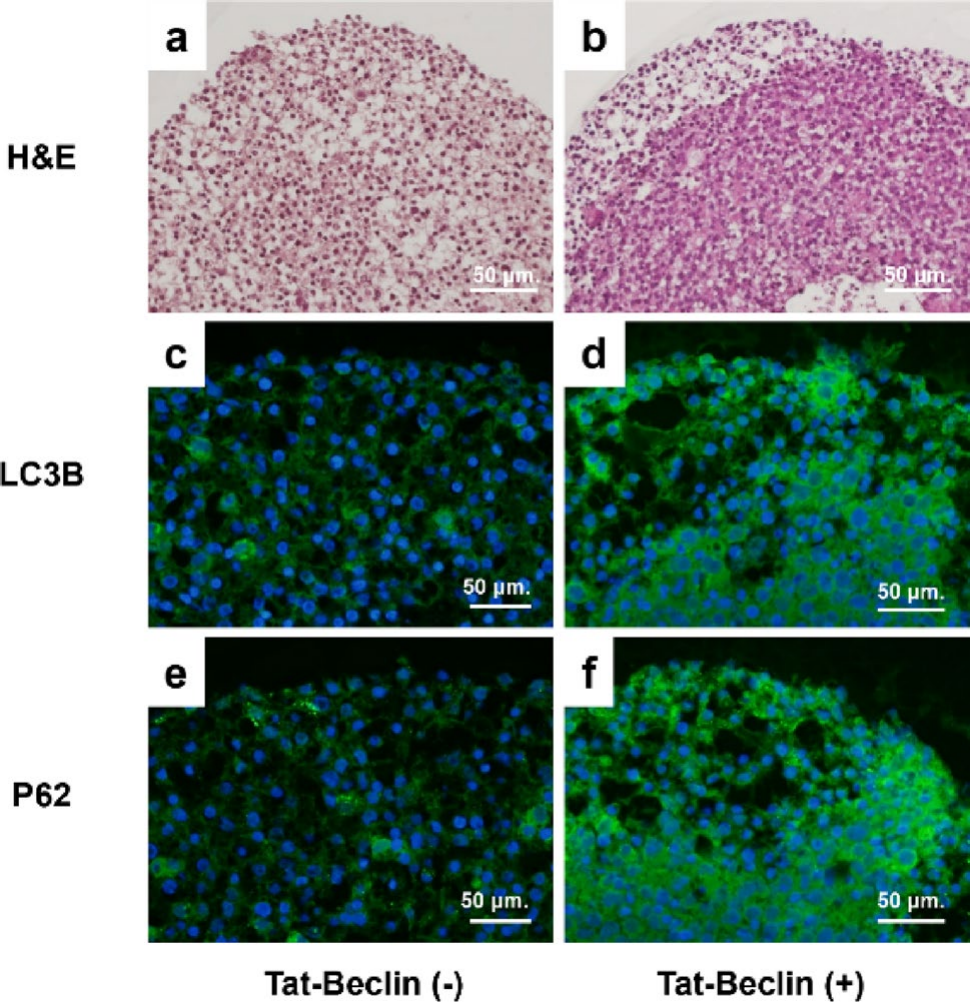
Effects of Tat-Beclin1 peptide on FKD spheroids. Treatment with Tat-Beclin1 D-11 peptide (20 µM) increased the proportion of non-vacuolated cells in FKD spheroids (**a**, **b**). Immunohistochemical analysis demonstrated that these cells with reduced lipid droplet accumulation were LC3B-positive and exhibited altered p62 distribution (**c**–**f**). These findings indicate a partial restoration of autophagic activity, although p62 accumulation was not fully normalized

Tat-Beclin1 treatment markedly increased the population of FKD spheroid cells without vacuolar degeneration (Fig. 7a, b). These cells showed strong LC3B immunoreactivity (Fig. 7c, d), while p62/SQSTM1 exhibited altered distribution with residual accumulation (Fig. 7e, f), suggesting partial restoration of autophagic flux.

## Discussion

In the present study, we demonstrated that knockdown of FBXW7 in hepatocellular carcinoma (HCC) cells induces profound alterations in lipid metabolism, autophagy, and cellular morphology. By integrating biochemical analyses with detailed ultrastructural and three-dimensional morphological observations, we showed that loss of FBXW7 leads to lipid droplet accumulation, impaired autophagy, and characteristic vacuolar degeneration, particularly evident in spheroid cultures. These findings highlight a critical role of FBXW7 in maintaining hepatocellular homeostasis through coordinated regulation of lipid metabolism and autophagic degradation.

FBXW7 is a well-established substrate recognition component of the SCF ubiquitin ligase complex and has been reported to regulate multiple oncogenic and metabolic proteins, including sterol regulatory element-binding protein 1 (SREBP-1) [8]. Consistent with previous reports, we observed increased levels of both precursor and mature SREBP-1 following FBXW7 knockdown, accompanied by upregulation of lipogenic gene expression. These data support the concept that FBXW7 loss enhances lipogenesis through stabilization of SREBP-1. Importantly, our study extends these molecular observations by providing morphological evidence of lipid droplet accumulation and structural deterioration at both the cellular and spheroid levels.

In addition to enhanced lipogenesis, our data indicate that autophagy is impaired in FBXW7-deficient cells. Reduced LC3B expression and accumulation of p62, together with ultrastructural features indicative of autophagy dysfunction observed by transmission electron microscopy, support this interpretation. However, because classical autophagic flux assays using lysosomal inhibitors such as bafilomycin A1 or chloroquine were not performed [4, 9, 10], we cannot definitively determine whether autophagy induction, degradation, or both are affected. Notably, both LC3B-I and LC3B-II levels were reduced, suggesting that impaired autophagy induction may contribute to the observed phenotype. The partial restoration of LC3B expression and morphological features by Tat-Beclin1 further supports the involvement of defective autophagy, although this intervention did not fully normalize p62 levels.

Based on these observations, we propose a conceptual model in which FBXW7 knockdown primarily promotes lipid accumulation through SREBP-1–mediated enhancement of lipogenesis, while concomitant impairment of autophagy secondarily suppresses lipophagy. These two processes are likely to act cooperatively, forming a feed-forward mechanism that exacerbates lipid droplet accumulation and cellular degeneration. While the relative contribution of each pathway cannot be quantitatively resolved in the present study, this integrated framework provides a mechanistic basis for understanding the observed morphological alterations.

The Tat-Beclin1 experiments were included as an exploratory approach to assess whether autophagy induction could modify the FBXW7 knockdown phenotype. Although Tat-Beclin1 partially ameliorated lipid accumulation and morphological abnormalities in both two-dimensional and three-dimensional cultures, the rescue was incomplete, particularly with respect to p62 normalization. Therefore, these results should not be interpreted as evidence of therapeutic efficacy but rather as supportive evidence for a causal relationship between autophagy impairment and the observed cellular and structural changes.

From a pathological perspective, the morphological features observed in FBXW7-deficient cells—namely lipid droplet accumulation, vacuolar degeneration, and surface irregularities of spheroids observed by low-vacuum scanning electron microscopy (LV-SEM)—bear conceptual resemblance to changes reported in metabolically dysregulated and MASH-related human HCC, including reports describing lipid-rich or vacuolated phenotypes in experimental and human HCC tissues [5–7]. Although direct extrapolation from in vitro systems to human disease is limited, our findings suggest that disruption of the FBXW7–SREBP-1–autophagy axis may contribute to morphological heterogeneity in metabolically altered HCC. In this context, the present study provides a morphological framework that may aid in the interpretation of lipid-rich or vacuolated tumor phenotypes.

It should be noted that, despite clear evidence of autophagy impairment, transmission electron microscopy did not reveal definitive ultrastructural features characteristic of Mallory-Denk bodies, such as conspicuous filamentous aggregates, nor did it show marked endoplasmic reticulum dilation indicative of overt endoplasmic reticulum stress. Accordingly, the involvement of Mallory-Denk body formation or ER stress cannot be concluded based on the present data. Further studies incorporating immunohistochemical or molecular analyses of stress-related markers will be required to address these possibilities [11, 12].

Several limitations of this study should be acknowledged. First, the analyses were performed using a single HCC cell line, Huh-7, and cell line–specific characteristics may influence lipid metabolism and autophagy [13]. Validation in additional HCC cell lines and normal hepatocyte models will be necessary to generalize these findings. Second, rescue experiments using siRNA-resistant FBXW7 were not conducted, and off-target effects of FBXW7 knockdown cannot be completely excluded. Finally, the lack of autophagic flux assays limits precise mechanistic interpretation of the autophagy defects observed.

In conclusion, our study demonstrates that FBXW7 knockdown disrupts hepatocellular homeostasis by coordinately altering lipid metabolism and autophagy, resulting in distinct morphological abnormalities. By combining molecular analyses with high-resolution morphological approaches, this work underscores the value of morphological evaluation in dissecting metabolic and autophagic dysregulation in HCC and provides a morphological and mechanistic foundation for future studies addressing the role of FBXW7 in metabolically dysregulated liver cancer.
